# Decoding Imagined Speech from EEG Data: A Hybrid Deep Learning Approach to Capturing Spatial and Temporal Features

**DOI:** 10.3390/life14111501

**Published:** 2024-11-18

**Authors:** Yasser F. Alharbi, Yousef A. Alotaibi

**Affiliations:** Computer Engineering Department, King Saud University, Riyadh 11451, Saudi Arabia; yaalotaibi@ksu.edu.sa

**Keywords:** neuroimaging, EEG, imagined speech, brain maps, topographic image

## Abstract

Neuroimaging is revolutionizing our ability to investigate the brain’s structural and functional properties, enabling us to visualize brain activity during diverse mental processes and actions. One of the most widely used neuroimaging techniques is electroencephalography (EEG), which records electrical activity from the brain using electrodes positioned on the scalp. EEG signals capture both spatial (brain region) and temporal (time-based) data. While a high temporal resolution is achievable with EEG, spatial resolution is comparatively limited. Consequently, capturing both spatial and temporal information from EEG data to recognize mental activities remains challenging. In this paper, we represent spatial and temporal information obtained from EEG signals by transforming EEG data into sequential topographic brain maps. We then apply hybrid deep learning models to capture the spatiotemporal features of the EEG topographic images and classify imagined English words. The hybrid framework utilizes a sequential combination of three-dimensional convolutional neural networks (3DCNNs) and recurrent neural networks (RNNs). The experimental results reveal the effectiveness of the proposed approach, achieving an average accuracy of 77.8% in identifying imagined English speech.

## 1. Introduction

Brain science has emerged as a crucial research field, particularly with recent advancements in neuroimaging methods, including functional magnetic resonance imaging (fMRI) and electroencephalography (EEG). These non-invasive tools enable us to record the neuronal activity of the brain and visualize its anatomy and function during various mental operations. This advancement has improved our comprehension of how the brain processes language, controls emotions, perceives stimuli, focuses attention, forms memories, and engages in decision making [1,2].

Different aspects of brain activity can be measured using non-invasive neuroimaging techniques, which are commonly categorized as either indirect or direct. fMRI is an indirect method that measures brain activity by detecting changes in blood flow and oxygen levels, known as the BOLD (blood–oxygen level-dependent) signal. This approach reflects neural activity indirectly through the hemodynamic response and requires a large and expensive device. This makes fMRI ideal for detailed brain mapping in cognitive research and clinical diagnosis [2,3].

In contrast, EEG is a widely used direct method that measures electrical activity by detecting voltage fluctuations from synchronized neuron firing. It captures the brain’s electrical activity from the scalp using multiple electrodes, providing real-time recordings of neural activity and offering important insights into brain function at different spatial and temporal resolutions [2,3]. This capability, combined with EEG’s portability and cost-effectiveness, makes it a powerful tool in neuroscience with potential applications in brain–computer interfaces. People can use these technologies to operate external devices, such as computer interfaces and prosthetic limbs [4]. Furthermore, EEG is utilized in clinical settings to diagnose and monitor various brain disorders. However, processing EEG signals presents challenges such as low signal-to-noise ratios, nonlinearity, and individual variability due to factors like age and psychological state [1,4].

In EEG-based recognition tasks, two key technical challenges often arise: the extraction of discriminative features from EEG signals and the development of effective computational models for accurate recognition. Traditionally, EEG classification has relied on hand-crafted features and classical machine learning techniques [1].

EEG features are usually extracted from four main domains: time, frequency, time–frequency, and spatial. Time-domain or temporal features capture signal values at specific time points or within time windows, while frequency-domain or spectral features measure signal power within defined frequency bands. Time-frequency features are obtained by analyzing the EEG signal in both the time and frequency domains simultaneously. Spatial features, on the other hand, focus on the spatial aspects of the signal, including the selection of relevant channels for specific tasks [5]. For example, in [6], time–frequency features were used to decode motor actions with a support vector machine. In contrast, another study [7] extracted features from all four domains and combined them with a source localization method to decode imagery intentions using an SVM-based model. While traditional methods have shown moderate success in decoding EEG, they require extensive domain knowledge to extract optimal features and often struggle with task generalization [4].

Recently, deep learning has been recognized as a significant tool in the fields of neuroimaging and brain monitoring/regulation. It has shown substantial potential for enhancing our understanding of brain function and advancing the development of brain–computer interfaces (BCIs) [4]. As a result, the application of deep learning algorithms to EEG-based tasks has been explored by many researchers. DL algorithms are structured into various architectural categories, including convolutional neural networks (CNNs), recurrent neural networks (RNNs), and hybrid neural network models. Among these architectures, CNNs have become the leading choice for EEG data classification tasks [8]. The use of CNNs for classification tasks involving imagined speech based on EEG signals has been widely investigated. Imagined speech involves mentally simulating speech without the physical movement of articulators or the production of sound [9,10,11].

Researchers have utilized various CNN-based techniques to enable the automatic learning of complex features and the classification of imagined speech from EEG signals. Research efforts in [12,13,14] explored various CNN-based methods for classifying imagined speech using raw EEG data or extracted features from the time domain.

Meanwhile, other studies have used images derived from EEG data as inputs for CNN-based models to recognize imagined speech. The study in [15] represented EEG data as two-dimensional images called scalograms, which combine time and frequency domains. The scalograms were then used as input for a CNN model with the purpose of classifying imagined words. The experimental results demonstrated that the CNN algorithm performed effectively when applied to scalogram images of EEG data.

Furthermore, in our previous work [16], we developed a method to classify imagined words using four CNN architectures. Each imagined word was represented by a single image consisting of sixteen EEG topographic brain maps, created from the time domain, to incorporate both spatial and temporal information from the EEG data. The study achieved effective classification accuracy, suggesting that topographic maps are a reliable method for distinguishing between different imagined speech patterns in EEG signals.

Although CNN-based models have demonstrated considerable success in classifying imagined speech from EEG signals, they are limited to capturing only local spatial features or short-term temporal patterns [17]. Simultaneously analyzing both the temporal and spatial aspects of EEG data is crucial, as it would provide a wealth of information concerning various brain states [18,19,20]. In this paper, we present a novel framework that employs a hybrid deep learning model based on three-dimensional (3D) CNNs and recurrent neural networks (RNNs) to effectively capture spatial and long-term dependencies in EEG data and subsequently classify imagined words. Our approach uses a sequence of topographic brain maps as input for the hybrid model, enabling the classification of distinct imagined words from EEG data.

## 2. Materials and Methods

**Figure 1:**
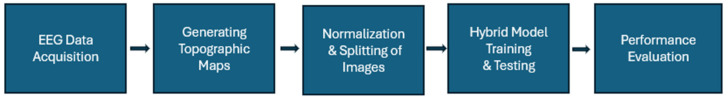
The general flowchart outlining the steps of our study.

The general flowchart representing the steps conducted in this study for imagined word classification using EEG signals is shown in Figure 1. The process begins with EEG Data Acquisition, where EEG signals of imagined speech are obtained from a public dataset. Next, in the Generating Topographic Maps step, the EEG signals are processed to create topographic brain images at regular intervals. These images are then Normalized and divided into training and testing datasets. In the Hybrid Model Training and Testing stage, a combination of 3D CNN and RNN architectures is created for model training and testing. Finally, in Performance Evaluation, accuracy metrics are calculated to evaluate the model’s effectiveness and reliability across different subjects.

**Figure 2:**
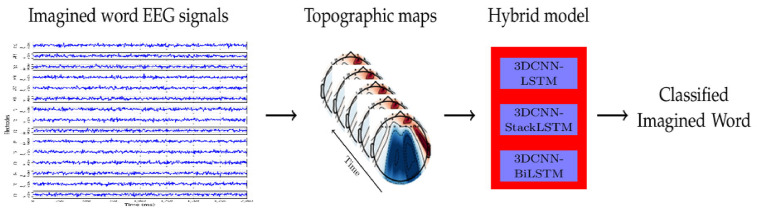
The proposed framework for identifying imagined words using EEG signals.

The framework proposed for classifying imagined speech based on EEG data is divided into three main components, as shown in Figure 2. It begins with raw EEG signals that have been recorded from multiple electrodes while a person imagines different words. In the second step, the signals are transformed into a sequence of topographic maps. Next, the topographic maps are processed according to a hybrid deep learning model that combines 3DCNNs with different RNNs architectures as a way to capture spatial and temporal features. Finally, the output from each model is employed to classify the imagined word. Each of these three steps is detailed below.

### 2.1. EEG Data Acquisition

The proposed method was evaluated using the publicly available BCI2020 dataset for imagined speech [21]. EEG data were collected from 15 participants using a BrainAmp device (Brain Products GmbH, Gilching, Germany) with a sampling rate of 256 Hz and 64 electrodes. All 64 channels correspond to the international 10–20 system. Each participant completed 70 trials, imagining five words (“Hello”, “Help me”, “Stop”, “Thank you”, and “Yes”). The participants were instructed to silently imagine pronouncing the given word without moving their articulators or producing any sound. All the participants were healthy and right-handed [14].

### 2.2. Transforming EEG Signals into Topographic Brain Maps

Topographic brain mapping is a common approach for spatially analyzing neural activity [22]. It visualizes EEG signals as images, offering insights into the brain’s structural and functional connectivity. It can be generated using raw EEG signals or features that have been extracted in the time or frequency domain. The topographic maps technique converts EEG data from a one-dimensional (1D) time series into either a two-dimensional (2D) or three-dimensional (3D) image, capturing both the spatial and temporal aspects of the EEG signal. This technique involves interpolating EEG signals from electrode locations to a grid of points, creating a colour-coded scalp map [12,23,24].

These topographic maps typically remain stable for about 60 to 120 ms before transitioning to different stable configurations. This stability period reflects the transient state of the brain’s overall neuronal activity [25,26].

In this study, we focused on EEG data from fifteen electrodes positioned at different regions on the frontal lobe of the brain (Fp1, AF3, Fp2, AF4, AF7, AF8, F1, Fz, F2, F7, F5, F3, F4, F6, and F8). We specifically selected electrodes covering the left and right frontal lobes due to the significant association between these regions and imagined speech, as previously demonstrated in our study [16] and in others [27,28].

To capture the majority of neuronal activity during each imagined speech trial in the BCI2020 dataset (a 2 s window), we chose 125 ms as the stable time duration, based on evidence suggesting that EEG topographic maps typically remain consistent for 60 to 120 ms [25,26]. This decision strikes a balance between maintaining a high temporal resolution and optimizing data management, reducing computational complexity while still capturing critical brain activity patterns.

**Figure 3:**
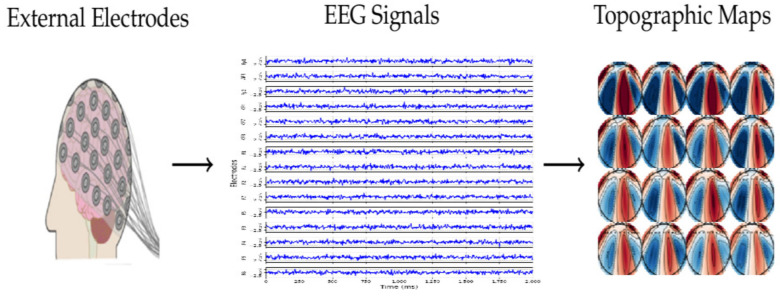
Transformation of EEG data into topographic maps [29].

In our experiment, during each imagined speech interval (2 s), the raw EEG signals from the fifteen electrodes for each trial were converted into two-dimensional topographic maps every 125 ms, with a slight 4 ms offset. This method generates 16 topographic maps per imagined speech task, providing a key representation of the brain’s dynamic responses throughout the task (see Figure 3).

The topographic maps generated for use in each experiment were divided into two segments; the first (80%) to train the deep learning model and the second (20%) to test the trained model, as detailed in Table 1. Prior to this division, the topographic images were normalized by scaling the pixel values to a range between 0 and 1 by dividing each pixel value by 255. This normalization step ensures consistent input data for the model training and testing.

We chose a training–testing split evaluation method over k-fold cross-validation because the dataset size was small, and training deep neural networks proved highly resource-intensive, due to the significant computational demands and large number of parameters involved [30].

### 2.3. Deep Learning Model

EEG signal processing pipelines are critical in effectively analyzing and interpreting EEG data for various applications. These pipelines can be categorized into two main types: (1) hybrid and (2) end-to-end. (1) Hybrid processing pipelines incorporate data preprocessing techniques, typically involving digital signal processing (DSP) methods, to eliminate artifacts and/or extract features before the EEG data are fed into machine learning models. (2) End-to-end processing pipelines employ only deep learning models, directly inputting raw EEG data without any preprocessing. Deep learning (DL) can automatically learn complex high-level and latent features from raw EEG signals through its deep architecture, effectively eliminating the need for time-intensive preprocessing and feature extraction steps [23,31,32].

In this study, we introduced a hybrid deep learning model combining 3D CNN and RNN techniques to classify imagined speech, based on topographic brain images from EEG data.

#### 2.3.1. Three-Dimensional Convolutional Neural Network (3D-CNN)

The convolutional neural network (CNN), also known as ConvNet, has become widely used as a powerful deep learning architecture and is particularly effective for handling high-dimensional data types, including images, videos, and EEG signals [33].

In CNNs, several types of convolutions are used to process different data types. Standard (2D) convolution is the most common and captures spatial features like edges and textures in 2D image data. Alternatively, 1D convolution operates across a single dimension and is appropriate for sequential data like time-series data, audio, or text. A further option is 3D convolution, which extends this concept into three dimensions, allowing it to capture both spatial and temporal features for classification tasks for videos, stacks of images, and 3D medical imaging [34].

**Figure 4:**
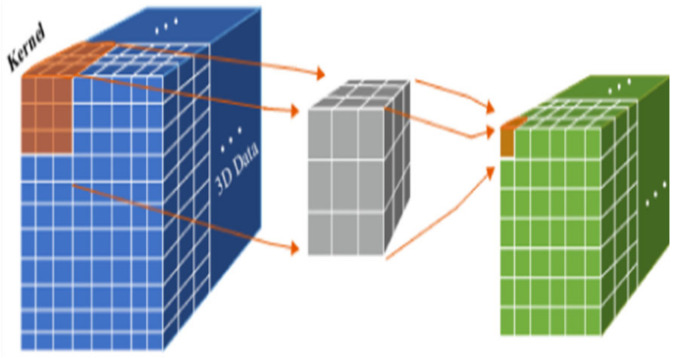
3D convolution procedure [35].

As shown in Figure 4, the 3D CNN architecture is capable of analyzing the position of objects over time using a 3D activation map throughout the convolution process, which makes it valuable for both data interpretation and capturing temporal context. The 3D convolution process uses a filter that shifts along three axes (*x*, *y*, *z*) to compute low-level feature representations. Each feature map location’s value can be computed using Equation (1). The resulting output forms a three-dimensional volume.

(1) $$V_{ij}^{xyz}=\tanh(b_{ij}+\sum_{m}\sum_{p=0}^{P_{i}-1}\sum_{q=0}^{Q_{i}-1}\sum_{r=0}^{R_{i}-1}w_{ijm}^{pqr}\;v_{\left(i-1\right)m}^{\left(x+p\right)\left(y+q\right)\left(z+r\right)})$$

where $V_{ij}^{xyz}$ is the output at position (*x*, *y*, *z*), $w_{ijm}^{pqr}$ represents the value of the kernel linked to the feature map from the preceding layer, $v_{\left(i-1\right)m}^{\left(x+p\right)\left(y+q\right)\left(z+r\right)}\;$ is the value from the previous layer’s feature map, $b_{ij}$ is the bias term, m is an index over the input feature maps from the previous layer, and $P_{i},\;Q_{i}$, $and\;R_{i}$ denote the Kernel size in the *x*, *y*, and *z* directions, respectively.

The functional maps are linked to record motion information; however, the convolution kernel is limited to extracting only one type of feature. The overall architecture of the network resembles that of a 2D convolutional neural network. Typically, similar to 2D convolution, better results can be achieved by merging several convolutional layers. The effectiveness of a 3D CNN relies on both the quantity of layers and the number and size of the filters within each layer. Although designed for 3D data, 3D convolutions can also be applied to 2D inputs such as images [35].

#### 2.3.2. Recurrent Neural Network (RNN)

A recurrent neural network (RNN) is a powerful type of neural network for managing sequence dependencies for text, audio, and video. A key characteristic of RNN architecture is its cyclic connections, enabling it to update its current state by considering both the present input data and prior states.

**Figure 5:**
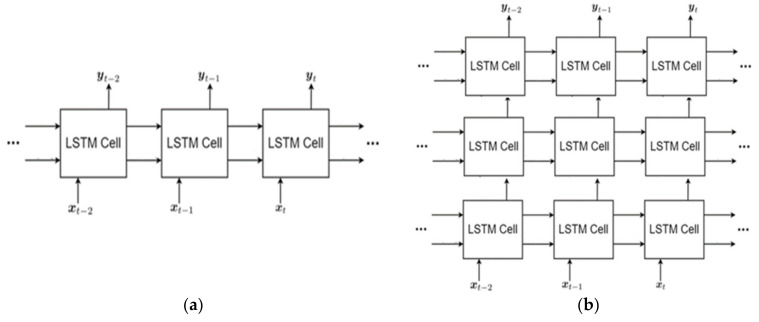
LSTM architectures: (**a**) simple LSTM; (**b**) stacked LSTM [37].

A popular variant of RNN is the Long Short-Term Memory (LSTM) network, introduced to address long-term dependencies in sequential data. The LSTM cell improves the memory capabilities of the traditional recurrent cell by implementing a “gate” mechanism within the cell [36]. As Figure 5a illustrates, LSTM consists of multiple cells, each corresponding to a specific time step. Within each LSTM cell, several gates serve the purpose of learning about different aspects of the input time series, helping the network manage and retain relevant information [37]. The operation of the LSTM block is governed by three gates: the input gate, the forget gate, and the output gate. Each of these gates determines the operations performed by the LSTM block when processing incoming inputs. At each time step, both the memory state and the output state are refreshed according to the following equations:

(2) $$\;i_{t}=\sigma (W_{i}\;\left[h_{t-1},x_{t}\right]+b_{i})$$

(3) $$f_{t}=\sigma (W_{f}\;\left[h_{t-1},x_{t}\right]+b_{f})$$

(4) $$c_{t}=f_{t}c_{t-1}+i_{t}tanh(W_{c}\;\left[h_{t-1},x_{t}\right]+b_{c})$$

(5) $$o_{t}=\sigma (W_{o}\;\left[h_{t-1},x_{t}\right]+b_{o})$$

(6) $$h_{t}=o_{t\;}\tanh\;(c_{t})\;$$

where $x_{t}$ is the input sequence, $h_{t}$ represents the output sequence, and $c_{t}$ indicates memory state at time *t*. Cell activations are denoted by c, and all values share the same dimensionality as the input vector. The input gate is represented by i, the forget gate by f, and the output gate by o. The symbol σ signifies the nonlinear sigmoid function, which is essential for determining gate activations.

The extension of the original LSTM is the stacked LSTM, which features multiple hidden LSTM layers, each containing several memory cells. As shown in Figure 5b, the stacked LSTM architecture comprises several LSTM layers arranged in a stack. Each layer analyzes the output from the preceding layer, enabling the network to understand more complex representations of the input series [37].

**Figure 6:**
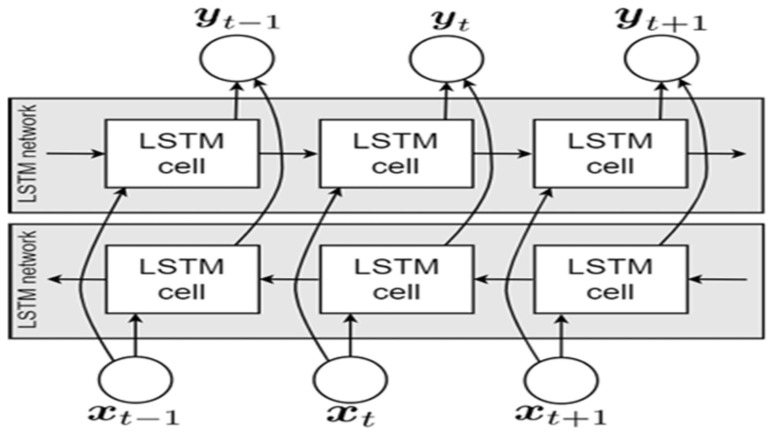
BiLSTM architecture.

Unlike traditional RNNs that process input sequences unidirectionally (either forward or backward), bidirectional LSTM (BiLSTM) networks are designed to capture information from both directions. This, therefore, gives them an enhanced capacity to understand context and dependencies within the data, making them particularly useful for tasks where context from both directions is essential. As illustrated in Figure 6, the BiLSTM architecture comprises two distinct LSTM layers: the forward LSTM layer, which processes the input sequence from beginning to finish, and the backward LSTM layer, which does the reverse [37]. The BiLSTM model consists of two LSTM layers that operate in both forward and backward directions, aiding in the detection of bidirectional long-term interdependencies between temporal phases. Therefore, the advantage of using BiLSTM is that features from both past and future time steps are included in the output [33].

#### 2.3.3. Proposed Hybrid Deep Learning Architectures

**Figure 7:**
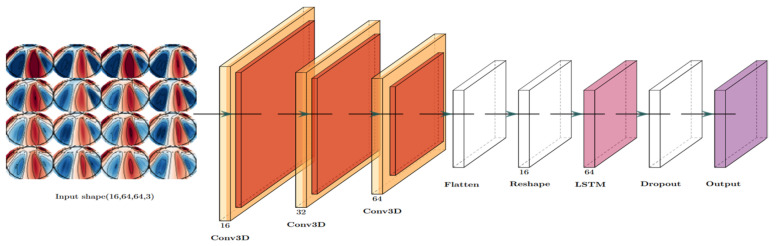
3DCNN-LSTM architecture.

As mentioned earlier, EEG signal analysis faces challenges such as low signal-to-noise ratios, nonlinearity, and individual variability. However, DL models have the potential to capture whole-brain dynamic information and leverage time-varying functional connectivity profiles, offering a promising avenue for advancing our understanding of brain function and disorders [38]. Our proposed architectures aim to extract both temporal and spatial features from sequences of topographic brain maps generated from raw EEG data to differentiate between various imagined words. We present hybrid models that integrate a 3DCNN for capturing spatial features from each topographic image and an RNN model for extracting both spatial and temporal patterns to process sequential topographic images. In this study, we propose three hybrid models as follows:

1. 3DCNN-LSTM model:

The structure of the proposed 3DCNN-LSTM model is presented in Figure 7. It begins by processing the input data, comprising 16 topographic maps, each being a 64 × 64 image with 3 color channels. The model first employs a 3D convolutional neural network (CNN), designed for spatial feature extraction. The first convolutional layer applies 16 filters with a 3 × 3 × 3 kernel, using ReLU activation and ‘same’ padding to preserve the spatial dimensions. This is followed by a 3D max pooling layer, with a 2 × 2 × 2 pool size intended to reduce spatial dimensions and computational complexity. The second convolutional layer increases the filters to 32, again using a 3 × 3 × 3 kernel with ReLU activation and ‘same’ padding, followed by another 2 × 2 × 2 max pooling layer. A third convolutional layer of 64 filters continues this pattern, and a final max pooling layer is then applied to improve the extraction of essential spatial characteristics.

After the 3D CNN layers, the output is flattened into a 1D vector in preparation for the next phase. The flattened data are then reshaped into a 2D sequence with 16 timesteps, making it suitable for processing in a long short-term memory (LSTM) network, which captures temporal dependencies. The LSTM layer has 64 units and outputs a single value after processing the full sequence, as return_sequences are set to a False value. To mitigate overfitting, a dropout layer with a rate of 40% is incorporated following the LSTM.

Finally, the output layer, a fully connected (dense) layer with softmax activation, generates a probability distribution for the number of classes, enabling the model to perform binary or multi-class classification. This architecture combines the strengths of the 3D CNN for spatial feature extraction and LSTM for temporal pattern recognition, making it suitable for imagined word classification using topographic brain maps including EEG data.

2. 3DCNN-StackLSTM model:

In this model, we replace the standard LSTM shown in Figure 7 with a dual LSTM network. The stacked LSTM layers in the 3DCNN-Stacked LSTM model are essential for capturing the complex temporal patterns and dependencies in the sequential data derived from topographic brain maps. The first LSTM layer is configured with 64 units and is set to return sequences, allowing it to output a sequence of hidden states for each input timestep. This is essential for enabling the subsequent LSTM layer to process the entire sequence of outputs so that each unit learns temporal relationships within the data while managing information flows through its gating mechanisms. The second LSTM layer, also consisting of 64 units, is configured not to return sequences, thereby compressing the output of the first layer into a single hidden state representing the entire input sequence. This design is beneficial for classification tasks and for capturing higher-level temporal features and interactions.

3. 3DCNN-BiLSTM model:

In this model, we replace the standard LSTM (shown in Figure 7) with a BiLSTM layer. The BiLSTM layer is a crucial component for temporal feature extraction after the spatial features have been captured by the preceding 3D CNN layers. This layer is configured with 64 units and is designed to process the reshaped 3D output of the CNN, which has been adjusted to fit the BiLSTM’s expected input format in terms of timesteps and features.

By processing the input sequence of 16 timesteps in both forward and backward directions, the BiLSTM effectively captures contextual information taken from both past and future images of the topographic sequence. This dual perspective allows the model to leverage the temporal dynamics of the data more comprehensively, making it especially powerful for classifying sequences of topographic brain images. Since the return_sequences parameter is set to False, the BiLSTM outputs a single vector to encapsulate the temporal context of the entire input sequence, which is then passed to the dropout layer for regularization prior to being fed into the output layer for classification. This design enhances the capacity of the model to recognize complex patterns over time.

## 3. Results

We implemented our proposed method using Python 3.10 and TensorFlow 2.15. All training and evaluation experiments were performed using NVIDIA GeForce RTX 2080 Ti GPU (NVIDIA Corporation, Santa Clara, CA, USA). The Adam optimizer was employed for training the models, utilizing a learning rate of 0.0001 and a batch size of 32 consistently across all experiments.

We formulated the challenge of categorizing imagined words as a supervised classification problem. In this context, to represent the imagined speech, the input was the sequence of sixteen topographic images of EEG data and the target output was the imagined word.

The efficiency of the deployed model will be evaluated using a subject-dependent approach, where each participant’s data are individually utilized for both training and testing. The average accuracy metric will be utilized to assess the effectiveness of the three hybrid deep learning models in this study, as it is the most commonly used experimental score in various EEG-based imagined speech research [8,39]. Each model’s accuracy will be measured for each subject using Equation (7), and then the overall average accuracy across all subjects will be computed using Equation (8).

(7) $$Accuracy=\frac{Number\;of\;correct\;predictions}{Total\;number\;of\;predictions}$$

(8) $$Average\;Accuracy=\frac{\sum_{s=1}^{N}{Accuracy}_{s}}{N}$$

where *N* indicates the number of subjects.

The experiment ensured equal representation of each class by balancing the participants’ trial counts. Several tasks were conducted using three hybrid deep learning models for word-pair and multi-class classification.

### 3.1. Word-Pair Classification Results

We categorized our experiments for the word-pair classification task according to word length as follows:

1. Short Imagined Words Classification:

In this experiment, we employed EEG topographic maps for three imagined words from the BCI2020 dataset—Yes, Stop, and Hello—to perform word-pair classification using three hybrid models.

**Figure 8:**
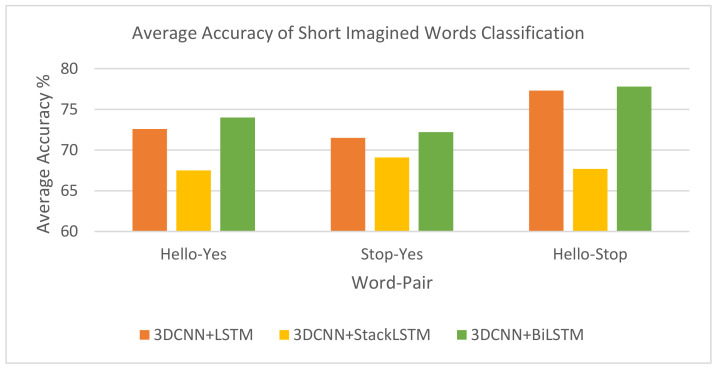
Average accuracy of short-word classification using three hybrid models.

Figure 8 and Table 2 present the average performance of three model architectures—3DCNN-LSTM (Model 1), 3DCNN-StackLSTM (Model 2), and 3DCNN-BiLSTM (Model 3) when classifying EEG topographic images for the three-word pairs: Hello–Yes, Stop–Yes, and Hello–Stop. The 3DCNN-BiLSTM model consistently outperforms the others, achieving the highest average scores across all pairs: 74% for Hello–Yes, 72.2% for Stop–Yes, and 77.8% for Hello–Stop. In contrast, the 3DCNN-StackLSTM model shows the lowest performance. The 3DCNN-LSTM model performs in the middle, showing competitive results, especially for the Hello–Stop pair (77.3%).

In addition, Subject #4 achieved the highest accuracy for the ‘Hello–Yes’ task regardless of the model architecture used, while Subject #8 had the highest classification accuracy for the ‘Hello–Stop’ task, also regardless of the model architecture. However, for the ‘Stop–Yes’ classification task, Subject #3 achieved the highest accuracy using the 3DCNN-StackLSTM model. This variation in performance is likely attributable to individual differences in brain signal characteristics and the ability of each model to extract key features from EEG data for each subject.

2. Long Imagined Phrases Classification:

In this experiment, we used the EEG topographic maps for two long imagined phrases from the BCI2020 dataset—Thank you and Help me—to conduct the word-pair classification with the three hybrid models.

**Figure 9:**
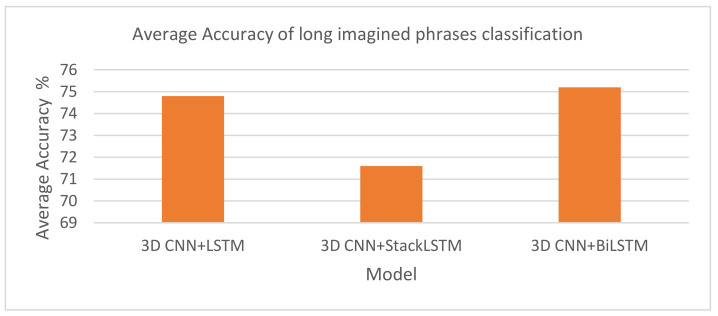
Average accuracy of long imagined phrase classification using three models.

Figure 9 and Table 3 show the average accuracy when classifying the imagined phrases “Help me” and “Thank you” using three different hybrid models. The results indicate that the 3DCNN-BiLSTM model was the most effective, achieving the highest average accuracy of 75.2% when classifying the words “Help me” and “Thank you”. Its design allows for the processing of input sequences from both forward and backward perspectives, which proved advantageous when leveraging contextual information. Similarly, the 3DCNN-LSTM model followed closely, with an average accuracy of 74.8%. This model demonstrated a strong capability to capture both the spatial and temporal features essential for accurate classification. In contrast, the 3DCNN-StackLSTM model exhibited a competitive performance with an average accuracy of 71.6% but was the least effective of the three architectures.

Comparing the results based on subjects’ performance, it is clear from Table 3 that Subject #3 achieved the highest accuracy with the 3DCNN-BiLSTM model (86.79%), while Subject #7 achieved the highest accuracy with the 3DCNN-StackLSTM model (85.36%). Additionally, Subject #12 achieved the highest accuracy with the 3DCNN-StackLSTM model (86.43%). The results of other subjects showed more variability in each model’s performance, highlighting the diverse nature of EEG data and the need for customized classification strategies for each subject.

3. Long–Short Imagined Words Classification:

In this experiment, we utilized EEG topographic maps of a mixture of imagined words from categories 1 and 2 to perform imagined word-pair classification using three hybrid models.

**Figure 10:**
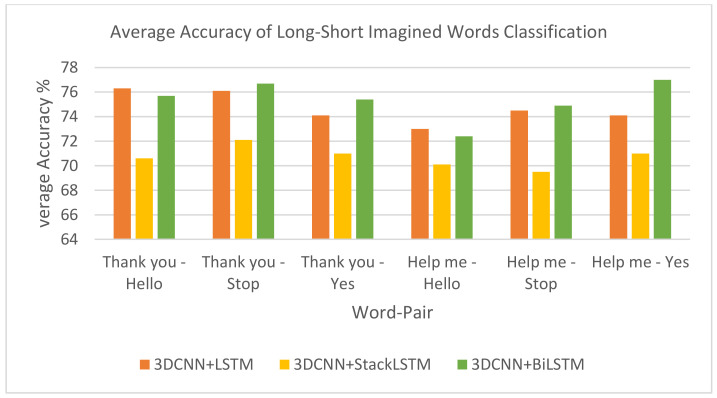
Average accuracy of long–short imagined words classification.

Table 4 and Table 5 and Figure 10 represent the performance results of three different hybrid models—across two sets of imagined word pairs: “Thank You–Hello”, “Thank you–Stop”, and “Thank you–Yes”, and “Help me–Hello”, “Help me–Stop”, and “Help me–Yes”.

The performance results across the two sets of word pairs demonstrate that the 3DCNN-BiLSTM model consistently delivers the highest average performance in all cases, achieving averages of 76.7% for the ‘Thank you–Stop’ word pair and 77.0% for the ‘Help me–Yes’ word pair. 3DCNN + LSTM follows closely behind, providing a solid middle-ground performance, often close to 3DCNN-BiLSTM.

On the other hand, 3DCNN-StackLSTM consistently exhibits the lowest average scores, falling behind the other two models across all word pairs.

In the overall results of word-pair classification tasks, the 3DCNN-BiLSTM model consistently showed the best performance across all tasks, while the 3DCNN-StackLSTM model generally underperformed compared to the other two models. Although all models demonstrated some effectiveness in classifying imagined words, performance varied significantly across subjects. This inconsistency highlights the influence of individual differences and the unique characteristics of EEG data on classification accuracy. Thus, the results emphasize the need for careful model selection based on each subject’s specific data.

### 3.2. Multi-Word Classification

In this experiment, we utilized EEG topographic maps of multiple imagined words to perform a multiword classification task using three hybrid models.

Table 6 reveals the experimental results from the three models (3D CNN-LSTM, 3D CNN-StackLSTM, and 3D CNN-BiLSTM) across multiple subjects to classify the five imagined words in the BCI2020 dataset, based on the sequence of EEG topographic maps. The results indicate the 3DCNN-StackLSTM model performs best overall, with an average score of 44.7%, outperforming both the 3DCNN-LSTM (40.7%) and the 3DCNN-BiLSTM (42.2%). Moreover, individual performance varied depending on the model used. The 3DCNN-LSTM model achieved the highest accuracy for Subject #7 (50.14%), while other models performed better for different subjects.

In addition, we investigated the performance of three different model architectures—3DCNN-LSTM, 3DCNN-StackLSTM, and 3DCNN-BiLSTM in the three short imagined word (Hello, Yes, and Stop) classification task.

Table 7 presents the average accuracy of the three models when classifying the three words across fifteen subjects. Among the models, the 3D CNN-Stack LSTM achieved the highest average accuracy of 59.5%, capturing the complex temporal dependencies within the data. The 3DCNN-BiLSTM followed closely, with an average accuracy of 59.2%, highlighting the impact of bidirectional processing when understanding contexts from both past and future time steps. In contrast, the 3DCNN-LSTM model recorded a lower average accuracy of 57.9%.

Furthermore, individual subject performance varied, with subject #4 yielding the highest accuracy across all the models; notably, in the 3DCNN-BiLSTM model, accuracy peaked at 70.95%. In contrast, Subject #5 showed the lowest accuracy across almost all the models. This indicates that the data from certain subjects may present greater challenges for the models to handle effectively.

In the overall results of multi-word classification tasks, the 3DCNN-StackLSTM model demonstrated good performance. Meanwhile, the 3D CNN-BiLSTM model also performed well, especially for some subjects. Also, the models exhibited diverse performance across subjects, indicating that the distinct characteristics of EEG data and individual differences created unique challenges for each model, especially in the multi-word classification task, where the difficulty increased

## 4. Discussion

The results of our experiments demonstrate that hybrid deep learning models can effectively classify imagined words from EEG topographic maps by capturing essential temporal and spatial features in EEG signals, even in the presence of typical challenges like low signal-to-noise ratios and individual variability. This approach shows promise for improving the accurate decoding of cognitive states based on neural data. Theoretically, these results provide valuable insights into the neural mechanisms underlying language processing, suggesting that hybrid models can capture complex neural dynamics associated with the generation and representation of imagined words. This advancement extends our understanding of functional brain connectivity in cognitive tasks and holds promise for several applications.

Our study’s results in imagined word-pair classification demonstrate that the 3DCNN-BiLSTM model significantly outperformed other architectures across nearly all word pairs, underscoring its particular suitability for imagined speech decoding tasks. This result is grounded in the model’s unique ability to process EEG sequences bidirectionally through the BiLSTM, enabling it to capture both the preceding and succeeding context in the data. Such context is crucial in imagined speech, where neural signals are inherently nuanced and context-dependent.

Furthermore, our multi-word classification experiments indicate that the 3DCNN-StackLSTM model emerged as the top performer for multi-class classification tasks. The deeper architecture of the StackLSTM allows it to capture complex temporal dependencies and develop richer sequential representations, which are crucial for distinguishing between multiple imagined words.

Practically, the 3DCNN-BiLSTM model’s enhanced capacity to interpret EEG signals for both short and long imagined word pairs suggests that it is a robust choice for several applications where linguistic variability in length and structure is common. Furthermore, its effectiveness in processing diverse imagined word types could enhance both medical and non-medical applications that rely on the precise decoding of imagined speech.

Theoretically, these findings underscore the advantage of using complex architectures for EEG data that involve intricate temporal dependencies. Although simpler models can be competitive in some cases, they often lack the depth required to fully capture these dependencies, particularly in context-sensitive tasks like imagined speech.

To further illustrate the comparative performance of the models, Table 8 sets out the training times for each model for the different classification tasks. The results show that training times increase with the complexity of the classification tasks. Among the architectures, 3DCNN-StackLSTM required the longest training time. This was due to the additional layers in the stacked LSTM, which introduce more parameters and complexity. In comparison, 3DCNN-LSTM and 3DCNN-BiLSTM required shorter training times, with the bidirectional LSTM needing more than the unidirectional counterpart due to the bi-directional data processing.

Although the 3DCNN-StackLSTM had the highest computational cost, it might offer improved performance for more complex multi-classification tasks by capturing richer temporal dependencies. Meanwhile, 3DCNN-BiLSTM balances the trade-off between model complexity and training time, affording a slight increase in time for potentially enhanced feature extraction. The 3DCNN-LSTM, despite being the fastest, may be better suited to simpler tasks where computational efficiency is a priority.

According to [1], the brain consists of numerous neurons whose activity generates distinct electrical potentials on the scalp that vary according to the level of alertness, responses to external stimuli, and other individual-specific factors. The results reveal that the individual differences among subjects significantly influenced the classification outcomes. This variability highlights the importance of accounting for individual characteristics when designing and evaluating EEG-based models, as each subject’s unique patterns and signal characteristics can pose challenges that impact the models’ ability to generalize effectively across different individuals.

Comparing our findings with recent studies on imagined word recognition using EEG data is difficult due to several factors, including the differences in data acquisition protocols, participant numbers, the variety and type of imagined speech words, and the classification algorithms used. Nevertheless, Table 9 provides a comparison of average accuracies derived from several recent works.

The table indicates that the majority of the utilized datasets are private, with the exceptions being the KaraOne database (KaraOne DB), the Pressel Coretto database (Pressel. DB), and the BCI Competition database (BCI DB), which are publicly available. Additionally, the number of subjects in each dataset varied significantly, ranging from as few as one subject to as many as fifteen (our work). The intervals for the imagined word trials also differed greatly, ranging from 1 s to 5 s. It is well known that longer durations of repeated word imagination can enhance model accuracy, although this renders the system less feasible as a tool for real-world use [10,39]. Taking these factors into account, our study represents a significant improvement in performance, as measured by average accuracy, in comparison to other leading methods.

## 5. Conclusions

This study proposes a hybrid deep learning framework for classifying imagined speech from EEG signals by extracting spatial and temporal features from topographic brain maps. Three hybrid deep learning models were applied and evaluated using the average accuracy metric.

Our findings highlight the effectiveness of combining 3DCNN and RNN models for EEG-based imagined speech classification. The 3DCNN-BiLSTM model achieved the highest average accuracy of 77.8% in word-pair tasks, while the 3DCNN-StackLSTM performed best in multi-class classification. While the models showed promising results, the variability between subjects remains a significant challenge. These findings suggest practical applications for brain–computer interface development and emphasize the theoretical need for personalized models that can adapt to individual EEG characteristics, enhancing the reliability and accuracy of imagined speech decoding.

This study presents several limitations. The limited dataset size may restrict the model’s ability to generalize across diverse EEG patterns, impacting performance consistency. Additionally, using only fifteen electrodes from the frontal lobe could reduce spatial information capture, potentially overlooking regions of the brain that may also be involved in imagined speech processing. Subject variability also presents a challenge, as individual differences in EEG patterns can affect model performance. Future work could benefit from expanded electrode coverage, larger datasets, and tailored approaches to account for individual EEG characteristics to improve generalizability.

## Figures and Tables

**Table 1:** Data division for model training and testing.

Experiment	Train	Test	Total
Word-pair classification	112 (80%)	28 (20%)	140
3-class classification	168 (80%)	42 (20%)	210
5-class classification	280 (80%)	70 (20%)	350

**Table 2:** Average accuracy of binary classification for short imagined words using three models.

Word-Pair	Hello–Yes			Stop–Yes			Hello–Stop		
Subject\Model *	1	2	3	1	2	3	1	2	3
1	72.5	70	78.57	72.5	59.29	71.43	87.14	75.36	86.79
2	71.79	64.64	74.29	68.21	68.57	70	79.64	68.57	73.57
3	65	63.57	66.43	76.79	**83.57**	82.14	74.64	71.79	76.79
4	**83.93**	**80.71**	**82.5**	73.93	68.21	67.14	75.36	68.21	75.36
5	73.93	65	71.07	71.79	66.07	72.86	76.43	55	78.21
6	72.86	73.93	79.64	68.93	71.79	71.79	77.86	67.14	75.71
7	66.07	68.57	64.64	77.5	71.07	73.93	74.29	68.21	75.36
8	79.29	72.5	78.21	**82.5**	79.29	**82.5**	**87.86**	**80.71**	**87.14**
9	68.21	63.57	69.29	71.43	66.43	71.07	71.43	70.36	70.71
10	75.71	65.36	75.71	61.79	63.93	66.43	68.93	67.5	71.79
11	76.06	66.79	77.86	66.79	68.93	72.5	77.86	57.14	76.06
12	67.14	63.57	68.57	62.86	62.5	60.71	68.57	66.79	68.21
13	65.36	61.79	68.93	70.36	65.36	71.07	80.36	59.64	85.36
14	80	68.21	80	68.93	69.29	69.64	78.93	70.36	83.93
15	71.07	64.29	73.93	78.57	72.5	79.29	80.71	68.21	81.43
Average	72.6	67.5	74	71.5	69.1	72.2	77.3	67.7	**77.8**

* 3DCNN + LSTM (1), 3DCNN + StackLSTM (2), 3DCNN + BiLSTM (3), Max = boldfaced, Min = underlined.

**Table 3:** Average accuracy of long imagined phrase classification using three models.

Subject\Model	3DCNN + LSTM	3DCNN + StackLSTM	3DCNN + BiLSTM
1	69.64	67.14	68.21
2	64.64	68.93	71.79
3	85.36	77.86	**86.79**
4	75.71	64.29	73.93
5	68.57	70	70.71
6	75.36	79.64	77.5
7	82.14	**85.36**	83.21
8	78.57	75	77.14
9	69.64	73.93	64.29
10	72.86	68.21	74.64
11	77.14	64.64	75.71
12	**86.43**	78.21	87.5
13	68.21	63.57	66.43
14	66.07	66.07	68.21
15	81.07	70.71	81.79
Average	74.8	71.6	**75.2**

Max = boldfaced, Min = underlined.

**Table 4:** Average accuracy of binary classification for ‘Thank You’ set words using three models.

Word-Pair	Thank You–Hello			Thank You–Stop			Thank You–Yes		
Subject\Model *	1	2	3	1	2	3	1	2	3
1	70	68.21	69.64	70.71	64.64	72.14	68.57	65	70
2	84.67	75.21	82.86	79.29	75.36	77.14	74.64	71.07	70.71
3	77.5	75.71	78.93	82.14	**81.43**	83.57	76.79	74.64	82.86
4	83.21	74.64	**90.36**	83.21	80.36	86.43	84.29	70	86.07
5	72.5	66.07	75	80	66.43	76.07	68.21	69.29	75.71
6	72.86	75.71	70.36	77.14	67.14	78.21	76.07	77.5	75.71
7	73.57	67.14	73.93	78.57	72.14	77.86	73.21	75	73.21
8	76.79	73.57	75.71	**89.29**	80.36	**89.29**	**87.14**	**79.64**	**86.79**
9	76.79	62.93	74.64	59.64	64.64	58.57	69.64	67.5	66.43
10	64.29	65.71	62.5	72.86	69.64	76.79	68.93	67.14	71.43
11	81.79	70	80.36	75.36	72.14	73.93	77.5	75	76.07
12	77.86	70.36	76.79	62.5	70.71	67.5	63.57	66.79	67.14
13	78.93	69.29	77.14	74.29	71.07	73.21	71.79	72.14	75.71
14	68.57	65.71	62.5	73.57	69.64	77.5	78.21	71.07	76.43
15	**85.36**	**79.29**	84.29	83.57	75.36	82.14	73.21	63.93	77.14
Average	76.3	70.6	75.7	76.1	72.1	**76.7**	74.1	71	75.4

* 3DCNN + LSTM (1), 3DCNN + StackLSTM (2), 3DCNN + BiLSTM (3), Max = boldfaced, Min = underlined.

**Table 5:** Average accuracies of binary classification for ‘Help me’ set words using three models.

Word-Pair	Help Me–Hello			Help Me–Stop			Help Me–Yes		
Subject\Model *	1	2	3	1	2	3	1	2	3
1	78.57	69.64	77.86	77.5	73.93	74.64	68.57	65	77.14
2	**84.64**	75.71	81.43	78.21	69.64	79.29	74.64	71.07	76.07
3	80.36	**77.5**	82.14	77.14	67.5	82.5	76.79	74.64	83.21
4	78.57	68.57	76.43	71.79	62.5	73.57	84.29	70	83.57
5	68.93	68.57	64.64	70.36	63.57	71.79	68.21	69.29	78.57
6	66.79	68.93	66.07	71.79	65	71.07	76.07	77.5	70.71
7	64.64	68.57	62.5	78.57	78.93	79.29	73.21	75	**84.29**
8	67.86	66.79	68.93	77.14	69.64	80.36	**87.14**	**79.64**	84.29
9	71.07	64.64	71.07	71.43	63.93	68.57	69.64	67.5	71.79
10	75	72.86	81.73	67.14	70.71	69.29	68.93	67.14	70
11	69.64	65	67.86	72.86	**79.29**	71.07	77.5	75	83.57
12	79.29	76.43	77.86	73.21	66.79	72.5	63.57	66.79	75.36
13	64.29	62.86	58.57	75	65	73.57	71.79	72.14	59.29
14	69.94	69.29	65.71	72.5	68.57	71.43	78.21	71.07	78.57
15	75.36	75.71	**83.57**	**83.57**	77.5	**84.64**	73.21	63.93	78.21
Average	73	70.1	72.4	74.5	69.5	74.9	74.1	71	**77**

* 3DCNN + LSTM (1), 3DCNN + StackLSTM (2), 3DCNN + BiLSTM (3), Max = boldfaced, Min = underlined.

**Table 6:** Average accuracy of multi-classification for five imagined words using three models.

Subject\Model	3D CNN + LSTM	3D CNN + StackLSTM	3D CNN + BiLSTM
1	41.57	44.43	41.29
2	41.14	48	42.71
3	43.29	48.29	45.57
4	47.43	49.43	48.43
5	37.14	41	39.57
6	41.43	49.43	42.71
7	**50.14**	**49.71**	**49.57**
8	43.43	43.57	43.29
9	40.43	42	41.71
10	34.14	41.14	40
11	45.29	48.57	43.14
12	34.71	41.71	39.86
13	33.57	36.57	34.29
14	43.14	47	43.43
15	33.71	39.71	38
Average	40.7	**44.7**	42.2

Max = boldfaced, Min = underlined.

**Table 7:** Averaged accuracies for three imagined word classifications using three models.

Subject\Model	3D CNN + LSTM	3D CNN + StackLSTM	3D CNN + BiLSTM
1	50.71	51.9	50.48
2	62.38	59.05	61.9
3	53.81	53.33	53.57
4	**67.38**	68.1	**70.95**
5	48.1	48.81	47.38
6	59.52	63.81	61.9
7	65.24	66.43	66.19
8	63.1	**68.57**	68.33
9	54.05	55.24	55
10	46.9	49.05	51.43
11	60.95	61.9	60.48
12	52.14	58.33	58.57
13	62.38	60.71	59.52
14	62.62	59.05	63.1
15	58.57	67.86	59.52
Average	57.9	**59.5**	59.2

Max = boldfaced, Min = underlined.

**Table 8:** Training times for different models and classification tasks.

Experiment\Model	3D CNN + LSTM	3DCNN + StackLSTM	3D CNN + BiLSTM
Word-pair classification	2.0 h *	2.3 h	2.1 h
3-class classification	3.4 h	4.5 h	3.7 h
5-class classification	4.8 h	7.7 h	6.2 h

* h (hour).

**Table 9:** Comparison of recent studies on the performance of imagined word decoding.

Paper	Dataset	Data Type	Model	Length (s)	Average Accuracy %	
					Word-Pair	Multiclass
[40]	12 Sub. * & 2 words	Private	Conv-attention	3	80.00	-
[41]	5 Sub. & 3 words	Private	ANN based	1	60.35	-
[27]	15 Sub. & 3 words	Private	SVM	3	-	50.10 (3-class)
[15]	13 Sub. & 5 words	Private	LSTM	2	-	73.56 (5-class)
[42]	-Pres.(15 Sub. & 6 words) -BCI (15 Sub. & 5 words)	Public	k-NN	Pres. (4) BCI (2)	-	45 Pres.(6-class) 48.10 BCI (5-lass)
[43]	Kara. (8 Sub. & 11words)	Public	SVM	5	85.3	81.6 (11-class)
[44]	One subject & 6 words	Private	RF & SVM	2	82.0	16.67 (6-class)
[45]	9 Sub. & 13 words	Private	SVM	1	75.56	46.54 (13-class)
[16]	BCI (15 Sub. & 5 words)	Public	CNN-based	2	76	59.7 (3-class)
Our work	BCI (15 Sub. & 5 words)	Public	3DCNN-RNNs	2	77.8	59.5 (3-class) 44.7 (5-class)

* Sub. (Subjects), Kara. (KaraOne DB), Pres. (Pressel. DB), BCI (BCI DB).

## Data Availability

The dataset used in this study is available at: https://osf.io/pq7vb (accessed on 30 September 2024).
